# The FGF metabolic axis

**DOI:** 10.1007/s11684-019-0711-y

**Published:** 2019-09-07

**Authors:** Xiaokun Li

**Affiliations:** School of Pharmaceutical Science, Wenzhou Medical University, Wenzhou, 325035 China

**Keywords:** FGF19, FGF21, FGF23, FGFR, metabolism, endocrine, Klotho

## Abstract

Members of the fibroblast growth factor (FGF) family play pleiotropic roles in cellular and metabolic homeostasis. During evolution, the ancestor FGF expands into multiple members by acquiring divergent structural elements that enable functional divergence and specification. Heparan sulfate-binding FGFs, which play critical roles in embryonic development and adult tissue remodeling homeostasis, adapt to an autocrine/paracrine mode of action to promote cell proliferation and population growth. By contrast, FGF19, 21, and 23 coevolve through losing binding affinity for extracellular matrix heparan sulfate while acquiring affinity for transmembrane α-Klotho (KL) or β-KL as a coreceptor, thereby adapting to an endocrine mode of action to drive interorgan crosstalk that regulates a broad spectrum of metabolic homeostasis. FGF19 metabolic axis from the ileum to liver negatively controls diurnal bile acid biosynthesis. FGF21 metabolic axes play multifaceted roles in controlling the homeostasis of lipid, glucose, and energy metabolism. FGF23 axes from the bone to kidney and parathyroid regulate metabolic homeostasis of phosphate, calcium, vitamin D, and parathyroid hormone that are important for bone health and systemic mineral balance. The significant divergence in structural elements and multiple functional specifications of FGF19, 21, and 23 in cellular and organismal metabolism instead of cell proliferation and growth sufficiently necessitate a new unified and specific term for these three endocrine FGFs. Thus, the term “FGF Metabolic Axis,” which distinguishes the unique pathways and functions of endocrine FGFs from other autocrine/paracrine mitogenic FGFs, is coined.

## References

[1] Beenken A, Mohammadi M (2009). The FGF family: biology, pathophysiology and therapy. Nat Rev Drug Discov.

[2] Luo Y, Ye S, Li X, Lu W (2019). Emerging structure-function paradigm of endocrine FGFs in metabolic diseases. Trends Pharmacol Sci.

[3] Li X, Wang C, Xiao J, McKeehan WL, Wang F (2016). Fibroblast growth factors, old kids on the new block. Semin Cell Dev Biol.

[4] Eriksson AE, Cousens LS, Weaver LH, Matthews BW (1991). Three-dimensional structure of human basic fibroblast growth factor. Proc Natl Acad Sci USA.

[5] Chen G, Liu Y, Goetz R, Fu L, Jayaraman S, Hu MC, Moe OW, Liang G, Li X, Mohammadi M (2018). αKlotho is a non-enzymatic molecular scaffold for FGF23 hormone signalling. Nature.

[6] Degirolamo C, Sabbà C, Moschetta A (2016). Therapeutic potential of the endocrine fibroblast growth factors FGF19, FGF21 and FGF23. Nat Rev Drug Discov.

[7] Luo Y, Ye S, Chen X, Gong F, Lu W, Li X (2017). Rush to the fire: FGF21 extinguishes metabolic stress, metaflammation and tissue damage. Cytokine Growth Factor Rev.

[8] McKeehan WL, Wang F, Kan M (1998). The heparan sulfate-fibroblast growth factor family: diversity of structure and function. Prog Nucleic Acid Res Mol Biol.

[9] Itoh N, Ornitz DM (2004). Evolution of the Fgf and Fgfr gene families. Trends Genet.

[10] Itoh N, Ornitz DM (2011). Fibroblast growth factors: from molecular evolution to roles in development, metabolism and disease. J Biochem.

[11] Luo Y, Lu W, Li X (2018). Unraveling endocrine FGF signaling complex to combat metabolic diseases. Trends Biochem Sci.

[12] Zhang X, Ibrahimi OA, Olsen SK, Umemori H, Mohammadi M, Ornitz DM (2006). Receptor specificity of the fibroblast growth factor family. The complete mammalian FGF family. J Biol Chem.

[13] Armelin HA (1973). Pituitary extracts and steroid hormones in the control of 3T3 cell growth. Proc Natl Acad Sci USA.

[14] Gospodarowicz D (1974). Localisation of a fibroblast growth factor and its effect alone and with hydrocortisone on 3T3 cell growth. Nature.

[15] Burgess WH, Maciag T (1989). The heparin-binding (fibroblast) growth factor family of proteins. Annu Rev Biochem.

[16] Luo Y, Ye S, Kan M, McKeehan WL (2006). Control of fibroblast growth factor (FGF) 7- and FGF1-induced mitogenesis and downstream signaling by distinct heparin octasaccharide motifs. J Biol Chem.

[17] Gospodarowicz D, Ill CR, Hornsby PJ, Gill GN (1977). Control of bovine adrenal cortical cell proliferation by fibroblast growth factor. Lack of effect of epidermal growth factor. Endocrinology.

[18] Mansour SL, Goddard JM, Capecchi MR (1993). Mice homozygous for a targeted disruption of the proto-oncogene int-2 have developmental defects in the tail and inner ear. Development.

[19] Guo C, Sun Y, Zhou B, Adam RM, Li X, Pu WT, Morrow BE, Moon A, Li X (2011). A Tbx1-Six1/Eya1-Fgf8 genetic pathway controls mammalian cardiovascular and craniofacial morphogenesis. J Clin Invest.

[20] Ornitz DM, Marie PJ (2015). Fibroblast growth factor signaling in skeletal development and disease. Genes Dev.

[21] Kan M, Wang F, Xu J, Crabb JW, Hou J, McKeehan WL (1993). An essential heparin-binding domain in the fibroblast growth factor receptor kinase. Science.

[22] Ye S, Luo Y, Lu W, Jones RB, Linhardt RJ, Capila I, Toida T, Kan M, Pelletier H, McKeehan WL (2001). Structural basis for interaction of FGF-1, FGF-2, and FGF-7 with different heparan sulfate motifs. Biochemistry.

[23] Goetz R, Mohammadi M (2013). Exploring mechanisms of FGF signalling through the lens of structural biology. Nat Rev Mol Cell Biol.

[24] Kouhara H, Hadari YR, Spivak-Kroizman T, Schilling J, Bar-Sagi D, Lax I, Schlessinger J (1997). A lipid-anchored Grb2-binding protein that links FGF-receptor activation to the Ras/MAPK signaling pathway. Cell.

[25] Huang Z, Marsiglia WM, Basu R U, Rahimi N, Ilghari D, Wang H, Chen H, Gai W, Blais S, Neubert TA, Mansukhani A, Traaseth NJ, Li X, Mohammadi M (2016). Two FGF receptor kinase molecules act in concert to recruit and transphosphorylate phospholipase Cγ. Mol Cell.

[26] Dorey K, Amaya E (2010). FGF signalling: diverse roles during early vertebrate embryogenesis. Development.

[27] Lu W, Luo Y, Kan M, McKeehan WL (1999). Fibroblast growth factor-10. A second candidate stromal to epithelial cell andromedin in prostate. J Biol Chem.

[28] Jin C, Wang F, Wu X, Yu C, Luo Y, McKeehan WL (2004). Directionally specific paracrine communication mediated by epithelial FGF9 to stromal FGFR3 in two-compartment premalignant prostate tumors. Cancer Res.

[29] Carter EP, Fearon AE, Grose RP (2015). Careless talk costs lives: fibroblast growth factor receptor signalling and the consequences of pathway malfunction. Trends Cell Biol.

[30] Goldberg JD, Zheng J, Castro-Malaspina H, Jakubowski AA, Heller G, van den Brink MR, Perales MA (2013). Palifermin is efficacious in recipients of TBI-based but not chemotherapy-based allogeneic hematopoietic stem cell transplants. Bone Marrow Transplant.

[31] Uchi H, Igarashi A, Urabe K, Koga T, Nakayama J, Kawamori R, Tamaki K, Hirakata H, Ohura T, Furue M (2009). Clinical efficacy of basic fibroblast growth factor (bFGF) for diabetic ulcer. Eur J Dermatol.

[32] Akita S, Akino K, Imaizumi T, Hirano A (2008). Basic fibroblast growth factor accelerates and improves second-degree burn wound healing. Wound Repair Regen.

[33] Fu X, Shen Z, Chen Y, Xie J, Guo Z, Zhang M, Sheng Z (1998). Randomised placebo-controlled trial of use of topical recombinant bovine basic fibroblast growth factor for second-degree burns. Lancet.

[34] Maddaluno L, Urwyler C, Werner S (2017). Fibroblast growth factors: key players in regeneration and tissue repair. Development.

[35] Zhao YZ, Zhang M, Wong HL, Tian XQ, Zheng L, Yu XC, Tian FR, Mao KL, Fan ZL, Chen PP, Li XK, Lu CT (2016). Prevent diabetic cardiomyopathy in diabetic rats by combined therapy of aFGFloaded nanoparticles and ultrasound-targeted microbubble destruction technique. J Control Release.

[36] Liang G, Song L, Chen Z, Qian Y, Xie J, Zhao L, Lin Q, Zhu G, Tan Y, Li X, Mohammadi M, Huang Z (2018). Fibroblast growth factor 1 ameliorates diabetic nephropathy by an anti-inflammatory mechanism. Kidney Int.

[37] Li R, Li Y Y, Zhao Y, Chen H, Yuan Y, Xu K, Zhang H, Lu Y, Wang J, Li X, Jia X, Xiao J (2018). Heparin-poloxamer thermosensitive hydrogel loaded with bFGF and NGF enhances peripheral nerve regeneration in diabetic rats. Biomaterials.

[38] Wu J, Zhu J, He C, Xiao Z, Ye J, Li Y, Chen A, Zhang H, Li X, Lin L, Zhao Y, Zheng J, Xiao J (2016). Comparative study of heparinpoloxamer hydrogel modified bFGF and aFGF for in vivo wound healing efficiency. ACS Appl Mater Interfaces.

[39] Wu J, Ye J, Zhu J, Xiao Z, He C, Shi H, Wang Y, Lin C, Zhang H, Zhao Y, Fu X, Chen H, Li X, Li L, Zheng J, Xiao J (2016). Heparin-based coacervate of FGF2 improves dermal regeneration by asserting a synergistic role with cell proliferation and endogenous facilitated VEGF for cutaneous wound healing. Biomacromolecules.

[40] Wang Q, He Y, Zhao Y, Xie H, Lin Q, He Z, Wang X, Li J, Zhang H, Wang C, Gong F, Li X, Xu H, Ye Q, Xiao J (2017). A thermosensitive heparin-poloxamer hydrogel bridges aFGF to treat spinal cord injury. ACS Appl Mater Interfaces.

[41] Katoh M (2016). Therapeutics targeting FGF signaling network in human diseases. Trends Pharmacol Sci.

[42] Liang G, Liu Z J, Cai Y, Li X (2012). Anticancer molecules targeting fibroblast growth factor receptors. Trends Pharmacol Sci.

[43] Cuevas P, Carceller F, Ortega S, Zazo M, Nieto I, Giménez-Gallego G (1991). Hypotensive activity of fibroblast growth factor. Science.

[44] Konishi M, Mikami T, Yamasaki M, Miyake A, Itoh N (2000). Fibroblast growth factor-16 is a growth factor for embryonic brown adipocytes. J Biol Chem.

[45] Rulifson IC, Collins P, Miao L, Nojima D, Lee KJ, Hardy M, Gupte J, Hensley K, Samayoa K, Cam C, Rottman JB, Ollmann M, Richards WG, Li Y (2017). *In vitro* and *in vivo* analyses reveal profound effects of fibroblast growth factor 16 as a metabolic regulator. J Biol Chem.

[46] Jonker JW, Suh JM, Atkins AR, Ahmadian M, Li P, Whyte J, He M, Juguilon H, Yin YQ, Phillips CT, Yu RT, Olefsky JM, Henry RR, Downes M, Evans RMA (2012). A PPARg-FGF1 axis is required for adaptive adipose remodelling and metabolic homeostasis. Nature.

[47] Huang Z, Tan Y, Gu J, Liu Y, Song L, Niu J, Zhao L, Srinivasan L, Lin Q, Deng J, Li Y, Conklin DJ, Neubert TA, Cai L, Li X, Mohammadi M (2017). Uncoupling the mitogenic and metabolic functions of FGF1 by tuning FGF1-FGF receptor dimer stability. Cell Reports.

[48] Badman MK, Pissios P, Kennedy AR, Koukos G, Flier JS, Maratos-Flier E (2007). Hepatic fibroblast growth factor 21 is regulated by PPARα and is a key mediator of hepatic lipid metabolism in ketotic states. Cell Metab.

[49] Inagaki T, Choi M, Moschetta A, Peng L, Cummins CL, McDonald JG, Luo G, Jones SA, Goodwin B, Richardson JA, Gerard RD, Repa JJ, Mangelsdorf DJ, Kliewer SA (2005). Fibroblast growth factor 15 functions as an enterohepatic signal to regulate bile acid homeostasis. Cell Metab.

[50] Inagaki T, Dutchak P, Zhao G, Ding X, Gautron L, Parameswara V, Li Y, Goetz R, Mohammadi M, Esser V, Elmquist JK, Gerard RD, Burgess SC, Hammer RE, Mangelsdorf DJ, Kliewer SA (2007). Endocrine regulation of the fasting response by PPARα-mediated induction of fibroblast growth factor 21. Cell Metab.

[51] Kharitonenkov A, Shiyanova TL, Koester A, Ford AM, Micanovic R, Galbreath EJ, Sandusky GE, Hammond LJ, Moyers JS, Owens RA, Gromada J, Brozinick JT, Hawkins ED, Wroblewski VJ, Li DS, Mehrbod F, Jaskunas SR, Shanafelt AB (2005). FGF-21 as a novel metabolic regulator. J Clin Invest.

[52] Goetz R, Ohnishi M, Ding X, Kurosu H, Wang L, Akiyoshi J, Ma J, Gai W, Sidis Y, Pitteloud N, Kuro OM, Razzaque MS, Mohammadi M. Klotho co-receptors inhibit signaling by paracrine FGF8 subfamily ligands. Mol Cell Biol 32(10):1944–1954 10.1128/MCB.06603-11 PMC3347405 22451487

[53] Luo Y, Yang C, Lu W, Xie R, Jin C, Huang P F, McKeehan WL (2010). Metabolic regulator βKlotho interacts with fibroblast growth factor receptor 4 (FGFR4) to induce apoptosis and inhibit tumor cell proliferation. J Biol Chem.

[54] Itoh M, Nacher JC, Kuma K, Goto S, Kanehisa M (2007). Evolutionary history and functional implications of protein domains and their combinations in eukaryotes. Genome Biol.

[55] Kurosu H, Choi M, Ogawa Y, Dickson AS, Goetz R, Eliseenkova AV, Mohammadi M, Rosenblatt KP, Kliewer SA, Kuro-o M (2007). Tissue-specific expression of βKlotho and fibroblast growth factor (FGF) receptor isoforms determines metabolic activity of FGF19 and FGF21. J Biol Chem.

[56] Fon Tacer K, Bookout AL, Ding X, Kurosu H, John GB, Wang L, Goetz R, Mohammadi M, Kuro-o M, Mangelsdorf DJ, Kliewer SA (2010). Research resource: comprehensive expression atlas of the fibroblast growth factor system in adult mouse. Mol Endocrinol.

[57] Wang H, Qiang L, Farmer SR (2008). Identification of a domain within peroxisome proliferator-activated receptor γ regulating expression of a group of genes containing fibroblast growth factor 21 that are selectively repressed by SIRT1 in adipocytes. Mol Cell Biol.

[58] Iizuka K, Takeda J, Horikawa Y (2009). Glucose induces FGF21 mRNA expression through ChREBP activation in rat hepatocytes. FEBS Lett.

[59] Wang Y, Solt LA, Burris TP (2010). Regulation of FGF21 expression and secretion by retinoic acid receptor-related orphan receptor α. J Biol Chem.

[60] Uebanso T, Taketani Y, Yamamoto H, Amo K, Tanaka S, Arai H, Takei Y, Masuda M, Yamanaka-Okumura H, Takeda E (2012). Liver X receptor negatively regulates fibroblast growth factor 21 in the fatty liver induced by cholesterol-enriched diet. J Nutr Biochem.

[61] Masuyama R, Stockmans I, Torrekens S, Van Looveren R, Maes C, Carmeliet P, Bouillon R, Carmeliet G (2006). Vitamin D receptor in chondrocytes promotes osteoclastogenesis and regulates FGF23 production in osteoblasts. J Clin Invest.

[62] Kolek OI, Hines ER, Jones MD, LeSueur LK, Lipko MA, Kiela PR, Collins JF, Haussler MR, Ghishan FK (2005). 1α,25-Dihydroxyvitamin D3 upregulates FGF23 gene expression in bone: the final link in a renal-gastrointestinal-skeletal axis that controls phosphate transport. Am J Physiol Gastrointest Liver Physiol.

[63] Zhang Y, Lei T, Huang JF, Wang SB, Zhou LL, Yang ZQ, Chen XD (2011). The link between fibroblast growth factor 21 and sterol regulatory element binding protein 1c during lipogenesis in hepatocytes. Mol Cell Endocrinol.

[64] Liu TF, Tang JJ, Li PS, Shen Y, Li JG, Miao HH, Li BL, Song BL (2012). Ablation of gp78 in liver improves hyperlipidemia and insulin resistance by inhibiting SREBP to decrease lipid biosynthesis. Cell Metab.

[65] Muise ES, Azzolina B, Kuo DW, El-Sherbeini M, Tan Y, Yuan X, Mu J, Thompson JR, Berger JP, Wong KK (2008). Adipose fibroblast growth factor 21 is up-regulated by peroxisome proliferatoractivated receptor γ and altered metabolic states. Mol Pharmacol.

[66] De Sousa-Coelho AL, Marrero PF, Haro D (2012). Activating transcription factor 4-dependent induction of FGF21 during amino acid deprivation. Biochem J.

[67] Yang C, Jin C, Li X, Wang F, McKeehan WL, Luo Y (2012). Differential specificity of endocrine FGF19 and FGF21 to FGFR1 and FGFR4 in complex with KLB. PLoS One.

[68] Lee S, Choi J, Mohanty J, Sousa LP, Tome F, Pardon E, Steyaert J, Lemmon MA, Lax I, Schlessinger J (2018). Structures of β-klotho reveal a ‘zip code’-like mechanism for endocrine FGF signalling. Nature.

[69] Luo Y, McKeehan WL (2013). Stressed liver and muscle call on adipocytes with FGF21. Front Endocrinol (Lausanne).

[70] Harrison SA, Rinella ME, Abdelmalek MF, Trotter JF, Paredes AH, Arnold HL, Kugelmas M, Bashir MR, Jaros MJ, Ling L, Rossi SJ, DePaoli AM, Loomba R (2018). NGM282 for treatment of nonalcoholic steatohepatitis: a multicentre, randomised, double-blind, placebo-controlled, phase 2 trial. Lancet.

[71] Hirschfield GM, Chazouillères O, Drenth JP, Thorburn D, Harrison SA, Landis CS, Mayo MJ, Muir AJ, Trotter JF, Leeming DJ, Karsdal MA, Jaros MJ, Ling L, Kim KH, Rossi SJ, Somaratne RM, DePaoli AM, Beuers U (2019). Effect of NGM282, an FGF19 analogue, in primary sclerosing cholangitis: a multicenter, randomized, double-blind, placebo-controlled phase II trial. J Hepatol.

[72] Harrison SA, Rossi SJ, Paredes AH, Trotter JF, Bashir MR, Guy CD, Banerjee R, Jaros MJ, Owers S, Baxter BA, Ling L, DePaoli AM. NGM282 improves liver fibrosis and histology in 12 weeks in patients with nonalcoholic steatohepatitis. Hepatology 2019 Feb 25. [Epub ahead of print] doi: 10.1002/hep.30590 10.1002/hep.30590 PMC7187438 30805949

[73] Sanyal A, Charles ED, Neuschwander-Tetri BA, Loomba R, Harrison SA, Abdelmalek MF, Lawitz EJ, Halegoua-DeMarzio D, Kundu S, Noviello S, Luo Y, Christian R (2018). Pegbelfermin (BMS-986036), a PEGylated fibroblast growth factor 21 analogue, in patients with non-alcoholic steatohepatitis: a randomised, doubleblind, placebo-controlled, phase 2a trial. Lancet.

[74] Talukdar S, Zhou Y, Li D, Rossulek M, Dong J, Somayaji V Y, Clark R, Lanba A, Owen BM, Brenner MB, Trimmer JK, Gropp KE, Chabot JR, Erion DM, Rolph TP, Goodwin B, Calle RA (2016). A long-acting FGF21 molecule, PF-05231023, decreases body weight and improves lipid profile in non-human primates and type 2 diabetic subjects. Cell Metab.

[75] Carpenter TO, Imel EA, Ruppe MD, Weber TJ, Klausner MA, Wooddell MM, Kawakami T, Ito T, Zhang X, Humphrey J, Insogna KL, Peacock M (2014). Randomized trial of the anti-FGF23 antibody KRN23 in X-linked hypophosphatemia. J Clin Invest.

[76] Yu C, Wang F, Kan M, Jin C, Jones RB, Weinstein M, Deng CX, McKeehan WL (2000). Elevated cholesterol metabolism and bile acid synthesis in mice lacking membrane tyrosine kinase receptor FGFR4. J Biol Chem.

[77] Fu L, John LM, Adams SH, Yu XX, Tomlinson E, Renz M, Williams PM, Soriano R, Corpuz R, Moffat B, Vandlen R, Simmons L, Foster J, Stephan JP, Tsai SP, Stewart TA (2004). Fibroblast growth factor 19 increases metabolic rate and reverses dietary and leptin-deficient diabetes. Endocrinology.

[78] Tomlinson E, Fu L, John L, Hultgren B, Huang X, Renz M, Stephan JP, Tsai SP, Powell-Braxton L, French D, Stewart TA (2002). Transgenic mice expressing human fibroblast growth factor-19 display increased metabolic rate and decreased adiposity. Endocrinology.

[79] Adams AC, Yang C, Coskun T, Cheng CC, Gimeno RE, Luo Y, Kharitonenkov A (2013). The breadth of FGF21’s metabolic actions are governed by FGFR1 in adipose tissue. Mol Metab.

[80] Walters JR, Tasleem AM, Omer OS, Brydon WG, Dew T, le Roux CW (2009). A new mechanism for bile acid diarrhea: defective feedback inhibition of bile acid biosynthesis. Clin Gastroenterol Hepatol.

[81] Oduyebo I, Camilleri M, Nelson AD, Khemani D, Nord SL, Busciglio I, Burton D, Rhoten D, Ryks M, Carlson P, Donato L, Lueke A, Kim K, Rossi SJ, Zinsmeister AR (2018). Effects of NGM282, an FGF19 variant, on colonic transit and bowel function in functional constipation: a randomized phase 2 trial. Am J Gastroenterol.

[82] Pai R, French D, Ma N, Hotzel K, Plise E, Salphati L, Setchell KD, Ware J, Lauriault V, Schutt L, Hartley D, Dambach D (2012). Antibodymediated inhibition of fibroblast growth factor 19 results in increased bile acids synthesis and ileal malabsorption of bile acids in cynomolgus monkeys. Toxicol Sci.

[83] Gerhard GS, Styer AM, Wood GC, Roesch SL, Petrick AT, Gabrielsen J, Strodel WE, Still CD, Argyropoulos G (2013). A role for fibroblast growth factor 19 and bile acids in diabetes remission after Roux-en-Y gastric bypass. Diabetes Care.

[84] Luo J, Ko B, Elliott M, Zhou M, Lindhout DA, Phung V, To C, Learned RM, Tian H, DePaoli AM, Ling L (2014). A nontumorigenic variant of FGF19 treats cholestatic liver diseases. Sci Transl Med.

[85] Schaap FG, van der Gaag NA, Gouma DJ, Jansen PL (2009). High expression of the bile salt-homeostatic hormone fibroblast growth factor 19 in the liver of patients with extrahepatic cholestasis. Hepatology.

[86] Benoit B, Meugnier E, Castelli M, Chanon S, Vieille-Marchiset A, Durand C, Bendridi N, Pesenti S, Monternier PA, Durieux AC, Freyssenet D, Rieusset J, Lefai E, Vidal H, Ruzzin J (2017). Fibroblast growth factor 19 regulates skeletal muscle mass and ameliorates muscle wasting in mice. Nat Med.

[87] Nicholes K, Guillet S, Tomlinson E, Hillan K, Wright B, Frantz GD, Pham TA, Dillard-Telm L, Tsai SP, Stephan JP, Stinson J, Stewart T, French DM (2002). A mouse model of hepatocellular carcinoma: ectopic expression of fibroblast growth factor 19 in skeletal muscle of transgenic mice. Am J Pathol.

[88] Zhou M, Learned RM, Rossi SJ, DePaoli AM, Tian H, Ling L (2016). Engineered fibroblast growth factor 19 reduces liver injury and resolves sclerosing cholangitis in Mdr2-deficient mice. Hepatology.

[89] Mayo MJ, Wigg AJ, Leggett BA, Arnold H, Thompson AJ, Weltman M, Carey EJ, Muir AJ, Ling L, Rossi SJ, DePaoli AM (2018). NGM282 for treatment of patients with primary biliary cholangitis: a multicenter, randomized, double-blind, placebo-controlled trial. Hepatol Commun.

[90] BonDurant LD, Potthoff MJ (2018). Fibroblast growth factor 21: a versatile regulator of metabolic homeostasis. Annu Rev Nutr.

[91] Giannini C, Feldstein AE, Santoro N, Kim G, Kursawe R, Pierpont B, Caprio S (2013). Circulating levels of FGF-21 in obese youth: associations with liver fat content and markers of liver damage. J Clin Endocrinol Metab.

[92] Lin Z, Gong Q, Wu C, Yu J, Lu T, Pan X, Lin S, Li X (2012). Dynamic change of serum FGF21 levels in response to glucose challenge in human. J Clin Endocrinol Metab.

[93] Yilmaz Y, Eren F, Yonal O, Kurt R, Aktas B, Celikel CA, Ozdogan O, Imeryuz N, Kalayci C, Avsar E (2010). Increased serum FGF21 levels in patients with nonalcoholic fatty liver disease. Eur J Clin Invest.

[94] Kliewer SA, Mangelsdorf DJ (2019). A dozen years of discovery: insights into the physiology and pharmacology of FGF21. Cell Metab.

[95] Laeger T, Henagan TM, Albarado DC, Redman LM, Bray GA, Noland RC, Münzberg H, Hutson SM, Gettys TW, Schwartz MW, Morrison CD (2014). FGF21 is an endocrine signal of protein restriction. J Clin Invest.

[96] Fisher FM, Kim M, Doridot L, Cunniff JC, Parker TS, Levine DM, Hellerstein MK, Hudgins LC, Maratos-Flier E, Herman MA (2017). A critical role for ChREBP-mediated FGF21 secretion in hepatic fructose metabolism. Mol Metab.

[97] von Holstein-Rathlou S, BonDurant LD, Peltekian L, Naber MC, Yin TC, Claflin KE, Urizar AI, Madsen AN, Ratner C, Holst B, Karstoft K, Vandenbeuch A, Anderson CB, Cassell MD, Thompson AP, Solomon TP, Rahmouni K, Kinnamon SC, Pieper AA, Gillum MP, Potthoff MJ (2016). FGF21 mediates endocrine control of simple sugar intake and sweet taste preference by the liver. Cell Metab.

[98] Talukdar S, Owen BM, Song P, Hernandez G, Zhang Y, Zhou Y, Scott WT, Paratala B, Turner T, Smith A, Bernardo B, Müller CP, Tang H, Mangelsdorf DJ, Goodwin B, Kliewer SA (2016). FGF21 regulates sweet and alcohol preference. Cell Metab.

[99] Fisher FM, Chui PC, Nasser IA, Popov Y, Cunniff JC, Lundasen T, Kharitonenkov A, Schuppan D, Flier JS, Maratos-Flier E (2014). Fibroblast growth factor 21 limits lipotoxicity by promoting hepatic fatty acid activation in mice on methionine and choline-deficient diets. Gastroenterology.

[100] Huang X, Yu C, Jin C, Yang C, Xie R, Cao D, Wang F, McKeehan WL (2006). Forced expression of hepatocyte-specific fibroblast growth factor 21 delays initiation of chemically induced hepatocarcinogenesis. Mol Carcinog.

[101] Tanaka N, Takahashi S, Zhang Y, Krausz KW, Smith PB, Patterson AD, Gonzalez FJ (2015). Role of fibroblast growth factor 21 in the early stage of NASH induced by methionine- and choline-deficient diet. Biochim Biophys Acta.

[102] Desai BN, Singhal G, Watanabe M, Stevanovic D, Lundasen T, Fisher FM, Mather ML, Vardeh HG, Douris N, Adams AC, Nasser IA, FitzGerald GA, Flier JS, Skarke C, Maratos-Flier E (2017). Fibroblast growth factor 21 (FGF21) is robustly induced by ethanol and has a protective role in ethanol associated liver injury. Mol Metab.

[103] Ye D Y, Li H, Jia W, Man K, Lo C Y, Lam KS, Xu A (2014). Fibroblast growth factor 21 protects against acetaminopheninduced hepatotoxicity by potentiating peroxisome proliferator-activated receptor coactivator protein-1α-mediated antioxidant capacity in mice. Hepatology.

[104] Singhal G, Kumar G, Chan S, Fisher FM, Ma Y, Vardeh HG, Nasser IA, Flier JS, Maratos-Flier E (2018). Deficiency of fibroblast growth factor 21 (FGF21) promotes hepatocellular carcinoma (HCC) in mice on a long term obesogenic diet. Mol Metab.

[105] Ye M, Lu W, Wang X, Wang C, Abbruzzese JL, Liang G, Li X, Luo Y (2016). FGF21-FGFR1 coordinates phospholipid homeostasis, lipid droplet function, and ER stress in obesity. Endocrinology.

[106] Foltz IN, Hu S, King C, Wu X, Yang C, Wang W, Weiszmann J, Stevens J, Chen JS, Nuanmanee N, Gupte J, Komorowski R, Sekirov L, Hager T, Arora T, Ge H, Baribault H F, Sheng J, Karow M, Wang M, Luo Y, McKeehan W, Wang Z, Véniant MM, Li Y (2012). Treating diabetes and obesity with an FGF21-mimetic antibody activating the βKlotho/FGFR1c receptor complex. Sci Transl Med.

[107] Gaich G, Chien JY, Fu H, Glass LC, Deeg MA, Holland WL, Kharitonenkov A, Bumol T, Schilske HK, Moller DE (2013). The effects of LY2405319, an FGF21 analog, in obese human subjects with type 2 diabetes. Cell Metab.

[108] Lin Z, Tian H, Lam KS, Lin S, Hoo RC, Konishi M, Itoh N, Wang Y, Bornstein SR, Xu A, Li X (2013). Adiponectin mediates the metabolic effects of FGF21 on glucose homeostasis and insulin sensitivity in mice. Cell Metab.

[109] Huang Z, Zhong L, Lee JTH, Zhang J, Wu D, Geng L, Wang Y, Wong CM, Xu A (2017). The FGF21–CCL11 axis mediates beiging of white adipose tissues by coupling sympathetic nervous system to type 2 immunity. Cell Metab.

[110] Lee P, Linderman JD, Smith S, Brychta RJ, Wang J, Idelson C, Perron RM, Werner CD, Phan GQ, Kammula US, Kebebew E, Pacak K, Chen KY, Celi FS (2014). Irisin and FGF21 are cold-induced endocrine activators of brown fat function in humans. Cell Metab.

[111] Hondares E, Iglesias R, Giralt A, Gonzalez FJ, Giralt M, Mampel T, Villarroya F (2011). Thermogenic activation induces FGF21 expression and release in brown adipose tissue. J Biol Chem.

[112] Ameka M, Markan KR, Morgan DA, BonDurant LD, Idiga SO, Naber MC, Zhu Z, Zingman LV, Grobe JL, Rahmouni K, Potthoff MJ (2019). Liver derived FGF21 maintains core body temperature during acute cold exposure. Sci Rep.

[113] Zhang Y, Xie Y, Berglund ED, Coate KC, He TT, Katafuchi T, Xiao G, Potthoff MJ, Wei W, Wan Y, Yu RT, Evans RM, Kliewer SA, Mangelsdorf DJ (2012). The starvation hormone, fibroblast growth factor-21, extends lifespan in mice. eLife.

[114] Youm YH, Horvath TL, Mangelsdorf DJ, Kliewer SA, Dixit VD (2016). Prolongevity hormone FGF21 protects against immune senescence by delaying age-related thymic involution. Proc Natl Acad Sci USA.

[115] Adams AC, Coskun T, Cheng CC, O’Farrell LS, Dubois SL, Kharitonenkov A (2013). Fibroblast growth factor 21 is not required for the antidiabetic actions of the thiazoladinediones. Mol Metab.

[116] Coate KC, Hernandez G, Thorne CA, Sun S, Le TDV, Vale K, Kliewer SA, Mangelsdorf DJ (2017). FGF21 is an exocrine pancreas secretagogue. Cell Metab.

[117] Singhal G, Fisher FM, Chee MJ, Tan TG, El O A, Adams AC, Najarian R, Kulkarni RN, Benoist C, Flier JS, Maratos-Flier E (2016). Fibroblast growth factor 21 (FGF21) protects against high fat diet induced inflammation and islet hyperplasia in pancreas. PLoS One.

[118] Johnson CL, Mehmood R, Laing SW, Stepniak CV, Kharitonenkov A, Pin CL (2014). Silencing of the fibroblast growth factor 21 gene is an underlying cause of acinar cell injury in mice lacking MIST1. Am J Physiol Endocrinol Metab.

[119] Johnson CL, Weston JY, Chadi SA, Fazio EN, Huff MW, Kharitonenkov A, Köester A, Pin CL (2009). Fibroblast growth factor 21 reduces the severity of cerulein-induced pancreatitis in mice. Gastroenterology.

[120] Kuroda M, Muramatsu R, Maedera N, Koyama Y, Hamaguchi M, Fujimura H, Yoshida M, Konishi M, Itoh N, Mochizuki H, Yamashita T (2017). Peripherally derived FGF21 promotes remyelination in the central nervous system. J Clin Invest.

[121] Soberg S, Sandholt CH, Jespersen NZ, Toft U, Madsen AL, von Holstein-Rathlou S, Grevengoed TJ, Christensen KB, Bredie WLP, Potthoff MJ, Solomon TPJ, Scheele C, Linneberg A, Jorgensen T, Pedersen O, Hansen T, Gillum MP, Grarup N (2017). FGF21 is a sugar-induced hormone associated with sweet intake and preference in humans. Cell Metab.

[122] Song P, Zechner C, Hernandez G, Canovas J, Xie Y, Sondhi V, Wagner M, Stadlbauer V, Horvath A, Leber B, Hu MC, Moe OW, Mangelsdorf DJ, Kliewer SA (2018). The hormone FGF21 stimulates water drinking in response to ketogenic diet and alcohol. Cell Metab.

[123] Frayling TM, Beaumont RN, Jones SE, Yaghootkar H, Tuke MA, Ruth KS, Casanova F, West B, Locke J, Sharp S, Ji Y, Thompson W, Harrison J, Etheridge AS, Gallins PJ, Jima D, Wright F, Zhou Y, Innocenti F, Lindgren CM, Grarup N, Murray A, Freathy RM, Weedon MN, Tyrrell J, Wood AR (2018). A common allele in FGF21 associated with sugar intake is associated with body shape, lower total body-fat percentage, and higher blood pressure. Cell Reports.

[124] Schumann G, Liu C, O’Reilly P, Gao H, Song P, Xu B, Ruggeri B, Amin N, Jia T, Preis S, Segura L M, Akira S, Barbieri C, Baumeister S, Cauchi S, Clarke TK, Enroth S, Fischer K, Hällfors J, Harris SE, Hieber S, Hofer E, Hottenga JJ, Johansson Å, Joshi PK, Kaartinen N, Laitinen J, Lemaitre R, Loukola A, Luan J, Lyytikäinen LP, Mangino M, Manichaikul A, Mbarek H, Milaneschi Y, Moayyeri A, Mukamal K, Nelson C, Nettleton J, Partinen E, Rawal R, Robino A, Rose L, Sala C, Satoh T, Schmidt R, Schraut K, Scott R, Smith AV, Starr JM, Teumer A, Trompet S, Uitterlinden AG, Venturini C, Vergnaud AC, Verweij N, Vitart V, Vuckovic D, Wedenoja J, Yengo L, Yu B, Zhang W, Zhao JH, Boomsma DI, Chambers J, Chasman DI, Daniela T, de G E, Deary I, Eriksson JG, Esko T, Eulenburg V, Franco OH, Froguel P, Gieger C, Grabe HJ, Gudnason V, Gyllensten U, Harris TB, Hartikainen AL, Heath AC, Hocking L, Hofman A, Huth C, Jarvelin MR, Jukema JW, Kaprio J, Kooner JS, Kutalik Z, Lahti J, Langenberg C, Lehtimäki T, Liu Y, Madden PA, Martin N, Morrison A, Penninx B, Pirastu N, Psaty B, Raitakari O, Ridker P, Rose R, Rotter JI, Samani NJ, Schmidt H, Spector TD, Stott D, Strachan D, Tzoulaki I, van der Harst P, van Duijn CM, Marques-Vidal P, Vollenweider P, Wareham NJ, Whitfield JB, Wilson J, Wolffenbuttel B, Bakalkin G, Evangelou E, Liu Y, Rice KM, Desrivières S, Kliewer SA, Mangelsdorf DJ, Müller CP, Levy D, Elliott P (2016). KLB is associated with alcohol drinking, and its gene product β-Klotho is necessary for FGF21 regulation of alcohol preference. Proc Natl Acad Sci USA.

[125] Restelli LM, Oettinghaus B, Halliday M, Agca C, Licci M, Sironi L, Savoia C, Hench J, Tolnay M, Neutzner A, Schmidt A, Eckert A, Mallucci G, Scorrano L, Frank S (2018). Neuronal mitochondrial dysfunction activates the integrated stress response to induce fibroblast growth factor 21. Cell Reports.

[126] Planavila A, Redondo I, Hondares E, Vinciguerra M, Munts C, Iglesias R, Gabrielli LA, Sitges M, Giralt M, van B M, Villarroya F (2013). Fibroblast growth factor 21 protects against cardiac hypertrophy in mice. Nat Commun.

[127] Morville T, Sahl RE, Trammell SA, Svenningsen JS, Gillum MP, Helge JW, Clemmensen C (2018). Divergent effects of resistance and endurance exercise on plasma bile acids, FGF19, and FGF21 in humans. JCI Insight.

[128] Brahma MK, Adam RC, Pollak NM, Jaeger D, Zierler KA, Pöcher N, Schreiber R, Romauch M, Moustafa T, Eder S, Ruelicke T, Preiss-Landl K, Lass A, Zechner R, Haemmerle G (2014). Fibroblast growth factor 21 is induced upon cardiac stress and alters cardiac lipid homeostasis. J Lipid Res.

[129] Lin Z, Pan F, Wu F, Ye D, Zhang Y, Wang Y, Jin L, Lian Q, Huang Y, Ding H, Triggle C, Wang K, Li X, Xu A (2015). Fibroblast growth factor 21 prevents atherosclerosis by suppression of hepatic sterol regulatory element-binding protein-2 and induction of adiponectin in mice. Circulation.

[130] Liu SQ, Roberts D, Kharitonenkov A, Zhang B, Hanson SM, Li YC, Zhang LQ, Wu YH (2013). Endocrine protection of ischemic myocardium by FGF21 from the liver and adipose tissue. Sci Rep.

[131] Yang H, Feng A, Lin S, Yu L, Lin X, Yan X, Lu X, Zhang C (2018). Fibroblast growth factor-21 prevents diabetic cardiomyopathy via AMPK-mediated antioxidation and lipid-lowering effects in the heart. Cell Death Dis.

[132] Zhang Chi, Huang Zhifeng, Gu Junlian, Yan Xiaoqing, Lu Xuemian, Zhou Shanshan, Wang Shudong, Shao Minglong, Zhang Fangfang, Cheng Peng, Feng Wenke, Tan Yi, Li Xiaokun (2015). Fibroblast growth factor 21 protects the heart from apoptosis in a diabetic mouse model via extracellular signal-regulated kinase 1/2-dependent signalling pathway. Diabetologia.

[133] Pan X, Shao Y, Wu F, Wang Y, Xiong R, Zheng J, Tian H, Wang B, Wang Y, Zhang Y, Han Z, Qu A, Xu H, Lu A, Yang T, Li X, Xu A, Du J, Lin Z (2018). FGF21 prevents angiotensin II–induced hypertension and vascular dysfunction by activation of ACE2/angiotensin-(1–7) axis in mice. Cell Metab.

[134] Kim KH, Jeong YT, Oh H, Kim SH, Cho JM, Kim YN, Kim SS, Kim DH, Hur KY, Kim HK, Ko T, Han J, Kim HL, Kim J, Back SH, Komatsu M, Chen H, Chan DC, Konishi M, Itoh N, Choi CS, Lee MS (2013). Autophagy deficiency leads to protection from obesity and insulin resistance by inducing Fgf21 as a mitokine. Nat Med.

[135] Suomalainen A, Elo JM, Pietiläinen KH, Hakonen AH, Sevastianova K, Korpela M, Isohanni P, Marjavaara SK, Tyni T, Kiuru-Enari  S, Pihko H, Darin N, Õunap K, Kluijtmans LA, Paetau A, Buzkova J, Bindoff LA, Annunen-Rasila J, Uusimaa J, Rissanen A, Yki-Järvinen H, Hirano M, Tulinius M, Smeitink J, Tyynismaa H (2011). FGF-21 as a biomarker for muscle-manifesting mitochondrial respiratory chain deficiencies: a diagnostic study. Lancet Neurol.

[136] Geng L, Liao B, Jin L, Huang Z, Triggle CR, Ding H, Zhang J, Huang Y, Lin Z, Xu A (2019). Exercise alleviates obesity-induced metabolic dysfunction via enhancing FGF21 sensitivity in adipose tissues. Cell Rep.

[137] Pereira RO, Tadinada SM, Zasadny FM, Oliveira KJ, Pires KMP, Olvera A, Jeffers J, Souvenir R, Mcglauflin R, Seei A, Funari T, Sesaki H, Potthoff MJ, Adams CM, Anderson EJ, Abel ED (2017). OPA1 deficiency promotes secretion of FGF21 from muscle that prevents obesity and insulin resistance. EMBO J.

[138] Tanimura Y, Aoi W, Takanami Y, Kawai Y, Mizushima K, Naito Y, Yoshikawa T (2016). Acute exercise increases fibroblast growth factor 21 in metabolic organs and circulation. Physiol Rep.

[139] Lee MS, Choi SE, Ha ES, An SY, Kim TH, Han SJ, Kim HJ, Kim DJ, Kang Y, Lee KW (2012). Fibroblast growth factor-21 protects human skeletal muscle myotubes from palmitate-induced insulin resistance by inhibiting stress kinase and NF-kB. Metabolism.

[140] Izumiya Y, Bina HA, Ouchi N, Akasaki Y, Kharitonenkov A, Walsh K (2008). FGF21 is an Akt-regulated myokine. FEBS Lett.

[141] Owen BM, Ding X, Morgan DA, Coate KC, Bookout AL, Rahmouni K, Kliewer SA, Mangelsdorf DJ (2014). FGF21 acts centrally to induce sympathetic nerve activity, energy expenditure, and weight loss. Cell Metab.

[142] Douris N, Stevanovic DM, Fisher FM, Cisu TI, Chee MJ, Nguyen NL, Zarebidaki E, Adams AC, Kharitonenkov A, Flier JS, Bartness TJ, Maratos-Flier E (2015). Central fibroblast growth factor 21 browns white fat via sympathetic action in male mice. Endocrinology.

[143] Liang Q, Zhong L, Zhang J, Wang Y, Bornstein SR, Triggle CR, Ding H, Lam KS, Xu A (2014). FGF21 maintains glucose homeostasis by mediating the cross talk between liver and brain during prolonged fasting. Diabetes.

[144] Owen BM, Bookout AL, Ding X, Lin VY, Atkin SD, Gautron L, Kliewer SA, Mangelsdorf DJ (2013). FGF21 contributes to neuroendocrine control of female reproduction. Nat Med.

[145] Bookout AL, de G M, Owen BM, Lee S, Gautron L, Lawrence HL, Ding X, Elmquist JK, Takahashi JS, Mangelsdorf DJ, Kliewer SA (2013). FGF21 regulates metabolism and circadian behavior by acting on the nervous system. Nat Med.

[146] Ishida N (2009). Role of PPARα in the control of torpor through FGF21-NPY pathway: from circadian clock to seasonal change in mammals. PPAR Res.

[147] Wang Q, Yuan J, Yu Z, Lin L, Jiang Y, Cao Z, Zhuang P, Whalen MJ, Song B, Wang XJ, Li X, Lo EH, Xu Y, Wang X (2018). FGF21 attenuates high-fat diet-induced cognitive impairment via metabolic regulation and anti-inflammation of obese mice. Mol Neurobiol.

[148] Yu Y, Bai F, Wang W, Liu Y, Yuan Q, Qu S, Zhang T, Tian G, Li S, Li D, Ren G (2015). Fibroblast growth factor 21 protects mouse brain against D-galactose induced aging via suppression of oxidative stress response and advanced glycation end products formation. Pharmacol Biochem Behav.

[149] Sarruf DA, Thaler JP, Morton GJ, German J, Fischer JD, Ogimoto K, Schwartz MW (2010). Fibroblast growth factor 21 action in the brain increases energy expenditure and insulin sensitivity in obese rats. Diabetes.

[150] Véniant MM, Hale C, Helmering J, Chen MM, Stanislaus S, Busby J, Vonderfecht S, Xu J, Lloyd DJ (2012). FGF21 promotes metabolic homeostasis via white adipose and leptin in mice. PLoS One.

[151] Xu J, Lloyd DJ, Hale C, Stanislaus S, Chen M, Sivits G, Vonderfecht S, Hecht R, Li YS, Lindberg RA, Chen JL, Jung DY, Zhang Z, Ko HJ, Kim JK, Véniant MM (2009). Fibroblast growth factor 21 reverses hepatic steatosis, increases energy expenditure, and improves insulin sensitivity in diet-induced obese mice. Diabetes.

[152] So WY, Cheng Q, Xu A, Lam KS, Leung PS (2015). Loss of fibroblast growth factor 21 action induces insulin resistance, pancreatic islet hyperplasia and dysfunction in mice. Cell Death Dis.

[153] Zhang C, Shao M, Yang H, Chen L, Yu L, Cong W, Tian H, Zhang F, Cheng P, Jin L, Tan Y, Li X, Cai L, Lu X (2013). Attenuation of hyperlipidemia- and diabetes-induced early-stage apoptosis and late-stage renal dysfunction via administration of fibroblast growth factor-21 is associated with suppression of renal inflammation. PLoS One.

[154] Kim HW, Lee JE, Cha JJ, Hyun YY, Kim JE, Lee MH, Song HK, Nam DH, Han JY, Han SY, Han KH, Kang YS, Cha DR (2013). Fibroblast growth factor 21 improves insulin resistance and ameliorates renal injury in *db/db* mice. Endocrinology.

[155] Tang TT, Li YY, Li JJ, Wang K, Han Y, Dong WY, Zhu ZF, Xia N, Nie SF, Zhang M, Zeng ZP, Lv BJ, Jiao J, Liu H, Xian ZS, Yang XP, Hu Y, Liao YH, Wang Q, Tu X, Mallat Z, Huang Y, Shi GP, Cheng X (2018). Liver-heart crosstalk controls IL-22 activity in cardiac protection after myocardial infarction. Theranostics.

[156] Wang N, Zhao TT, Li SM, Li YH, Wang YJ, Li DS, Wang WF (2019). Fibroblast growth factor 21 ameliorates pancreatic fibrogenesis via regulating polarization of macrophages. Exp Cell Res.

[157] Li S, Guo X, Zhang T, Wang N, Li J, Xu P, Zhang S, Ren G, Li D (2017). Fibroblast growth factor 21 ameliorates high glucose-induced fibrogenesis in mesangial cells through inhibiting STAT5 signaling pathway. Biomed Pharmacother.

[158] Li S, Wang N, Guo X, Li J, Zhang T, Ren G, Li D (2018). Fibroblast growth factor 21 regulates glucose metabolism in part by reducing renal glucose reabsorption. Biomed Pharmacother.

[159] Lin XL, He XL, Zeng JF, Zhang H, Zhao Y, Tan JK, Wang Z (2014). FGF21 increases cholesterol efflux by upregulating ABCA1 through the ERK1/2-PPARγ-LXRα pathway in THP1 macrophage-derived foam cells. DNA Cell Biol.

[160] Yu Y, He J, Li S, Song L, Guo X, Yao W, Zou D, Gao X, Liu Y, Bai F, Ren G, Li D (2016). Fibroblast growth factor 21 (FGF21) inhibits macrophage-mediated inflammation by activating Nrf2 and suppressing the NF-κB signaling pathway. Int Immunopharmacol.

[161] Li H, Wu G, Fang Q, Zhang M, Hui X, Sheng B, Wu L, Bao Y, Li P, Xu A, Jia W (2018). Fibroblast growth factor 21 increases insulin sensitivity through specific expansion of subcutaneous fat. Nat Commun.

[162] Li SM, Wang WF, Zhou LH, Ma L, An Y, Xu WJ, Li TH, Yu YH, Li DS, Liu Y (2015). Fibroblast growth factor 21 expressions in white blood cells and sera of patients with gestational diabetes mellitus during gestation and postpartum. Endocrine.

[163] Li JY, Wang N, Khoso MH, Shen CB, Guo MZ, Pang XX, Li DS, Wang WF (2018). FGF-21 elevated IL-10 production to correct LPS-induced inflammation. Inflammation.

[164] Wang WF, Ma L, Liu MY, Zhao TT, Zhang T, Yang YB, Cao HX, Han XH, Li DS (2015). A novel function for fibroblast growth factor 21: stimulation of NADPH oxidase-dependent ROS generation. Endocrine.

[165] Li SM, Yu YH, Li L, Wang WF, Li DS (2016). Treatment of CIA mice with FGF21 down-regulates TH17-IL-17 axis. Inflammation.

[166] Saito H, Kusano K, Kinosaki M, Ito H, Hirata M, Segawa H, Miyamoto K, Fukushima N (2003). Human fibroblast growth factor-23 mutants suppress Na^+^-dependent phosphate co-transport activity and 1α,25-dihydroxyvitamin D3 production. J Biol Chem.

[167] Shimada T, Kakitani M, Yamazaki Y, Hasegawa H, Takeuchi Y, Fujita T, Fukumoto S, Tomizuka K, Yamashita T (2004). Targeted ablation of Fgf23 demonstrates an essential physiological role of FGF23 in phosphate and vitamin D metabolism. J Clin Invest.

[168] Shimada T, Mizutani S, Muto T, Yoneya T, Hino R, Takeda S, Takeuchi Y, Fujita T, Fukumoto S, Yamashita T (2001). Cloning and characterization of FGF23 as a causative factor of tumor-induced osteomalacia. Proc Natl Acad Sci USA.

[169] Andrukhova O, Smorodchenko A, Egerbacher M, Streicher C, Zeitz U, Goetz R, Shalhoub V, Mohammadi M, Pohl EE, Lanske B, Erben RG (2014). FGF23 promotes renal calcium reabsorption through the TRPV5 channel. EMBO J.

[170] Andrukhova O, Slavic S, Smorodchenko A, Zeitz U, Shalhoub V, Lanske B, Pohl EE, Erben RG (2014). FGF23 regulates renal sodium handling and blood pressure. EMBO Mol Med.

[171] Ben-Dov IZ, Galitzer H, Lavi-Moshayoff V, Goetz R, Kuro-o M, Mohammadi M, Sirkis R, Naveh-Many T, Silver J (2007). The parathyroid is a target organ for FGF23 in rats. J Clin Invest.

[172] Toro L, Barrientos V, León P, Rojas M, Gonzalez M, González-Ibáñez A, Illanes S, Sugikawa K, Abarzúa N, Bascuñán C, Arcos K, Fuentealba C, Tong AM, Elorza AA, Pinto ME, Alzamora R, Romero C, Michea L (2018). Erythropoietin induces bone marrow and plasma fibroblast growth factor 23 during acute kidney injury. Kidney Int.

[173] Rabadi S, Udo I, Leaf DE, Waikar SS, Christov M (2018). Acute blood loss stimulates fibroblast growth factor 23 production. Am J Physiol Renal Physiol.

[174] ADHR Consortium. (2000). Autosomal dominant hypophosphataemic rickets is associated with mutations in FGF23. Nat Genet.

[175] Bowe AE, Finnegan R, Jan de Beur SM, Cho J, Levine MA, Kumar R, Schiavi SC (2001). FGF-23 inhibits renal tubular phosphate transport and is a PHEX substrate. Biochem Biophys Res Commun.

[176] Riminucci M, Collins MT, Fedarko NS, Cherman N, Corsi A, White KE, Waguespack S, Gupta A, Hannon T, Econs MJ, Bianco P, Gehron Robey P (2003). FGF-23 in fibrous dysplasia of bone and its relationship to renal phosphate wasting. J Clin Invest.

[177] Hoffman WH, Jueppner HW, Deyoung BR, O’dorisio MS, Given KS (2005). Elevated fibroblast growth factor-23 in hypophosphatemic linear nevus sebaceous syndrome. Am J Med Genet A.

[178] Kato K, Jeanneau C, Tarp MA, Benet-Pagès A, Lorenz-Depiereux B, Bennett EP, Mandel U, Strom TM, Clausen H (2006). Polypeptide GalNAc-transferase T3 and familial tumoral calcinosis. Secretion of fibroblast growth factor 23 requires O-glycosylation. J Biol Chem.

[179] Ichikawa S, Imel EA, Sorenson AH, Severe R, Knudson P, Harris GJ, Shaker JL, Econs MJ (2006). Tumoral calcinosis presenting with eyelid calcifications due to novel missense mutations in the glycosyl transferase domain of the GALNT3 gene. J Clin Endocrinol Metab.

[180] Garringer HJ, Fisher C, Larsson TE, Davis SI, Koller DL, Cullen MJ, Draman MS, Conlon N, Jain A, Fedarko NS, Dasgupta B, White KE (2006). The role of mutant UDP-N-acetyl-α-D-galactosamine-polypeptide N-acetylgalactosaminyltransferase 3 in regulating serum intact fibroblast growth factor 23 and matrix extracellular phosphoglycoprotein in heritable tumoral calcinosis. J Clin Endocrinol Metab.

[181] Benet-Pagès A, Orlik P, Strom TM, Lorenz-Depiereux B (2005). An FGF23 missense mutation causes familial tumoral calcinosis with hyperphosphatemia. Hum Mol Genet.

[182] Chefetz I, Heller R, Galli-Tsinopoulou A, Richard G, Wollnik B, Indelman M, Koerber F, Topaz O, Bergman R, Sprecher E, Schoenau E (2005). A novel homozygous missense mutation in FGF23 causes Familial Tumoral Calcinosis associated with disseminated visceral calcification. Hum Genet.

[183] Araya K, Fukumoto S, Backenroth R, Takeuchi Y, Nakayama K, Ito N, Yoshii N, Yamazaki Y, Yamashita T, Silver J, Igarashi T, Fujita T (2005). A novel mutation in fibroblast growth factor 23 gene as a cause of tumoral calcinosis. J Clin Endocrinol Metab.

[184] Abbasi F, Ghafouri-Fard S, Javaheri M, Dideban A, Ebrahimi A, Ebrahim-Habibi A (2014). A new missense mutation in FGF23 gene in a male with hyperostosis-hyperphosphatemia syndrome (HHS). Gene.

[185] Faul C, Amaral AP, Oskouei B, Hu MC, Sloan A, Isakova T, Gutiérrez OM, Aguillon-Prada R, Lincoln J, Hare JM, Mundel P, Morales A, Scialla J, Fischer M, Soliman EZ, Chen J, Go AS, Rosas SE, Nessel L, Townsend RR, Feldman HI, St J S M, Ojo A, Gadegbeku C, Di M G, Reuter S, Kentrup D, Tiemann K, Brand M, Hill JA, Moe OW, Kuro-O M, Kusek JW, Keane MG, Wolf M (2011). FGF23 induces left ventricular hypertrophy. J Clin Invest.

[186] Gutiérrez OM, Januzzi JL, Isakova T, Laliberte K, Smith K, Collerone G, Sarwar A, Hoffmann U, Coglianese E, Christenson R, Wang TJ, deFilippi C, Wolf M (2009). Fibroblast growth factor 23 and left ventricular hypertrophy in chronic kidney disease. Circulation.

[187] McGrath ER, Himali JJ, Levy D, Conner SC, Pase MP, Abraham CR, Courchesne P, Satizabal CL, Vasan RS, Beiser AS, Seshadri S (2019). Circulating fibroblast growth factor 23 levels and incident dementia: The Framingham heart study. PLoS One.

[188] Liu P, Chen L, Bai X, Karaplis A, Miao D, Gu N (2011). Impairment of spatial learning and memory in transgenic mice overexpressing human fibroblast growth factor-23. Brain Res.

